# The Voluntary Hospitals and the House of Commons

**Published:** 1911-08-05

**Authors:** 


### The Hospital

A Journal of

The Medical Sciences and Hospital Administration.

New Series. No. 236, Vol. IX. [No. 1308, Vol. L.] SATURDAY, AUGUST 5, 1911

### THE VOLUNTARY HOSPITALS AND THE HOUSE OF COMMONS.

The discussion in the House of Commons on Clause 12 of the Bill, as will be seen in another column, has dealt to some extent with the position of the voluntary hospitals. We have always been of opinion that it was undesirable to raise the position of the hospitals under this clause because the effect of what has been done, whilst it cannot provide the cost, or even a material portion of the cost of in-patient treatment for the assured affected by it, may excite a feeling of irritation against the hospitals on the part of those who have no dependants. Mr. Austen Chamberlain was quite right in stating that the Chancellor of the Exchequer's amendment, as carried, in no real sense will benefit the voluntary hospitals. It may indeed cause a good deal of additional pressure upon the in-patient accommodation by people who, if admitted, may have to be treated at a heavy loss to the hospitals. Now the voluntary hospitals cannot afford to do the work the State requires for the insured except on a *pro rata* basis. Mr. Lloyd George seems to think that he has dealt with the hospitals in a tentative way under Clause 12, and, as we understand him, that there he must leave them. If that indeed be his decision we hope that Mr. Chamberlain or some important member of the House will move the simple amendments to Clause 15 which we have advocated, whereby the Local Health Committees will be empowered to make arrangements with hospitals for the treatment of insured persons entitled to institutional benefit under the Act. If this be done Mr. Lloyd George will go down to history as a man who has displayed great statesmanship under difficult circumstances by providing the opportunity, through his Bill, to reorganise our whole system of medical treatment, whereby overlapping may be eradicated, and inter-communication and co-operation between all practitioners, the voluntary hospitals and sanitary authorities may be secured, to the benefit of the sick and the welfare of the whole of the people.

Mr. Lloyd George on Tuesday made it clear that he does not understand what in fact must happen to the voluntary hospitals if the Insurance Bill as it stands becomes law. We believe with the Chancellor of the Exchequer that the out-patient departments as they at present exist will soon disappear. In substitution for them every properly administered hospital will have a consultation branch and a big casualty department where applicants for relief will be seen once and then referred to a member of the medical profession or to a provident dispensary, or to the consultation department, as circumstances may be found to warrant. Mr. Lloyd George fails to realise that, because his Bill must effect changes greatly to the advantage of all who need hospital care and of the general practitioner, the relations between the hospitals and the profession must improve, and the consequent pressure upon hospital beds be much greater than ever before. This pressure will probably mean a considerable increase in the cost of in-patient treatment, because the additional cases are likely to be those requiring operative treatment and specially difficult cases, for reasons which Dr. Saleeby pointed out in the "Pall Mall Gazette" of August 1. Not only will the voluntary hospitals have to face this demand for increased expenditure by the hospital requirements of the insured, but the fall in the hospital's income, as we have shown in previous articles, must be considerable.

It is no answer to this point, as Mr. Lloyd George appears to think, to say that under this Bill the man who was paying 6d. to a friendly society will in future pay 4d. for sick benefits. Thus, argues Mr. Lloyd George, he would not only have the means but the best encouragement to give at least half of this 2d. to the hospital funds. The clear answer to this point, which Mr. Lloyd George has put forward under an evident misapprehension, is that the subscriptions to the hospitals from the working classes come for the most part from workmen who may not be members of a Friendly Society. The workmen's contributions to Friendly Societies and to the voluntary hospitals were voluntary, but the Bill will compel every workman to pay at least 4d. a week for insurance, and it is practically certain that when these payments for sickness are made compulsory upon all workers, the latter will consider that the former claim upon them for voluntary contributions may be disregarded. Great misapprehension is apparent upon this point, not only on the part of the Chancellor of the Exchequer, but of Members of the House of Commons, though it is one of vital importance to the financial strength of the voluntary hospitals.

Of course it is not possible to forecast exactly the effects of legislation such as that of the Insurance Bill upon individuals or upon indispensable institutions like the voluntary hospitals. For this reason we claim that statesmanship demands that the Insurance Bill shall provide, as it can readily do under Clause 15, that the hospitals shall be put upon the same footing as the new sanatoria which are to be created under the Bill. This can be readily done by amending Clause 15 so as to effect this result. Here let us say that those who have most to do with the treatment of tuberculosis are aware that the establishment of sanatoria may be overdone and is likely to be overdone if the funds granted for their erection at the outset are as large as the Bill, as drafted, contemplates. The sum set aside for sanatoria is ample to provide also for a *pro rata* payment to the voluntary hospitals for such insured persons as may require hospital treatment. Let Parliament beware of the danger of erecting all over the country a great number of sanatoria buildings which twenty-five years hence may in the whole or in part be left empty, because, as there is very good ground for believing, science may discover a remedy for tuberculosis which will render unnecessary the contemplated wholesale resort to open-air treatment which at the best is not a cure but a palliative.

We desire to help Mr. Lloyd George to make this Insurance Bill of the utmost practical value in reorganising the whole of our national health arrangements and place them once for all upon lines which are calculated to secure by inter-communication and co-operation the abolition of overlapping, of hospital abuse, and malingering. So we may build up a system which shall gradually become as efficient and helpful to the people as it is possible to provide. As the Medical Council has declared, and we agree, no voluntary hospital worthy of the name can or will object to Government inspection, a point which the Bill can provide for when the amendment to Clause 15 is made to include the administration of hospital as well as of sanatorium benefit. We do most earnestly urge the Chancellor of the Exchequer to concede the amendments to Clause 15 which we have ventured to suggest, because the health committees will then have it in their power to control the amount of hospital and sanatoria benefits which will be given to the insured. In this way we may guarantee that on a *pro rata* basis the principle of voluntary hospital support and administration shall be maintained, whilst the funds available for the creation of new sanatoria are economically and carefully administered, so as to avoid as far as possible the risk of erecting a number of expensive new buildings which may in a few years fail to be required for the work contemplated under the Act.

